# Relationship between perceptual learning in speech and statistical learning in younger and older adults

**DOI:** 10.3389/fnhum.2014.00628

**Published:** 2014-09-01

**Authors:** Thordis M. Neger, Toni Rietveld, Esther Janse

**Affiliations:** ^1^Centre for Language Studies, Radboud University NijmegenNijmegen, Netherlands; ^2^International Max Planck Research School for Language SciencesNijmegen, Netherlands; ^3^Donders Institute for Brain, Cognition and Behaviour, Radboud University NijmegenNijmegen, Netherlands

**Keywords:** perceptual learning, statistical learning, individual differences, aging, working memory, attention switching control, processing speed, vocabulary

## Abstract

Within a few sentences, listeners learn to understand severely degraded speech such as noise-vocoded speech. However, individuals vary in the amount of such perceptual learning and it is unclear what underlies these differences. The present study investigates whether perceptual learning in speech relates to statistical learning, as sensitivity to probabilistic information may aid identification of relevant cues in novel speech input. If statistical learning and perceptual learning (partly) draw on the same general mechanisms, then statistical learning in a non-auditory modality using non-linguistic sequences should predict adaptation to degraded speech. In the present study, 73 older adults (aged over 60 years) and 60 younger adults (aged between 18 and 30 years) performed a visual artificial grammar learning task and were presented with 60 meaningful noise-vocoded sentences in an auditory recall task. Within age groups, sentence recognition performance over exposure was analyzed as a function of statistical learning performance, and other variables that may predict learning (i.e., hearing, vocabulary, attention switching control, working memory, and processing speed). Younger and older adults showed similar amounts of perceptual learning, but only younger adults showed significant statistical learning. In older adults, improvement in understanding noise-vocoded speech was constrained by age. In younger adults, amount of adaptation was associated with lexical knowledge and with statistical learning ability. Thus, individual differences in general cognitive abilities explain listeners' variability in adapting to noise-vocoded speech. Results suggest that perceptual and statistical learning share mechanisms of implicit regularity detection, but that the ability to detect statistical regularities is impaired in older adults if visual sequences are presented quickly.

## Introduction

Listeners' ability to rapidly learn to understand unfamiliar speech conditions such as accented, disordered or noise-vocoded speech is impressive. Within a few sentences, listeners learn to map a new type of speech input onto their old percept, some improving their speech recognition performance by more than 60% (Eisner et al., 2010). However, listeners show great variability in the amount of such perceptual learning (Eisner et al., 2010). This raises the question which mechanisms underlie perceptual learning.

Perceptual learning can be defined as “relatively long-lasting changes to an organism's perceptual system that improve its ability to respond to its environment” (Goldstone, 1998, p. 585). As listeners are not able to describe the changes that led to their improved perception, perceptual learning is assumed to be a type of implicit learning (Fahle, 2006). A conceptual framework that accounts for changes in the perceptual system is the Reverse Hierarchy Theory (RHT) (Ahissar and Hochstein, 2004). The RHT argues that perceptual learning is a top–down guided process. When a listener is exposed to a novel speech condition, initial performance fails as the speech input can no longer be readily matched to higher-level representations such as word representations. According to the RHT, prolonged exposure modifies these higher-level representations, which subsequently enables top–down guidance to retune weights at lower levels of the processing hierarchy: the weights of task-relevant input are increased and the weights of task-irrelevant input are pruned. This process of weight retuning starts at the highest level of the hierarchy and continues gradually to the lower levels (i.e., the reverse hierarchy). When lower-level representations have been modified, performance under difficult conditions can be based on accessing these low-level representations. This is illustrated by findings that adaptation to noise-vocoded speech generalizes to novel words (Hervais-Adelman et al., 2008), to non-words (Loebach et al., 2008) and to the recognition of environmental sounds (Loebach et al., 2009). These generalization findings suggest that perceptual learning in speech modifies representations at lower levels of the hierarchy, that is, representations at a sublexical level (Hervais-Adelman et al., 2008; Banai and Amitay, 2012).

The RHT has been influential in explaining behavioral observations in visual and auditory perceptual learning (Nahum et al., 2010; Banai and Amitay, 2012; Cohen et al., 2013; Sabin et al., 2013). However, the RHT does not specify which processes take place in the initial stages of adaptation that enable the perceptual system to identify task-relevant cues in the input and to modify high-level representations. One of the basic principles in the RHT and other models of perceptual learning is the retuning of weights based on the relevance of features or dimensions for the specific task (Goldstone, 1998; Dosher and Lu, 1999; Ahissar and Hochstein, 2004; Petrov et al., 2005). This principle implies that stimuli have to share certain features, which can thus be considered task-relevant, for perceptual learning and for transfer of learning to occur. Accordingly, several studies have highlighted the importance of structural regularities (Cohen et al., 2013) and of stimulus consistencies for perceptual learning (e.g., Nahum et al., 2010). In other words, for learning to occur, participants need to detect specific regularities in the input. Therefore, individual differences in sensitivity to such regularities may indicate why listeners differ in adapting to unfamiliar speech input.

An implicit learning mechanism that has been linked to pattern sensitivity is statistical learning. Statistical or probabilistic learning describes the ability to implicitly extract regularities from an input by detecting the probabilities with which properties co-occur (Misyak and Christiansen, 2012). Statistical learning has gained increasing attention over the past years in language research, as language itself is probabilistic in nature (Auer and Luce, 2005). Accordingly, co-occurrence probabilities of units have been shown to facilitate processing at various linguistic levels (e.g., effects of phonotactic probability; Vitevitch et al., 2004) or transitional probability (e.g., Thompson and Newport, 2007). Statistical learning has been found to be of major importance in language acquisition (Saffran, 2003). Also in adulthood, individual differences in statistical learning have been shown to predict sentence processing performance (Misyak and Christiansen, 2012). Moreover, deficits in statistical learning ability have been reported for various language-related disorders such as specific language impairment (Evans et al., 2009), agrammatic aphasia (Christiansen et al., 2010), and language-based learning disabilities (Grunow et al., 2006). As statistical probabilities are provided and continuously updated by the input, relying on statistical probabilities actually enables language users to adapt to their environment, which is the essential characteristic of perceptual learning. Therefore, the present study aims to investigate whether statistical learning relates to perceptual learning in speech perception. If adaptation to a novel speech condition and statistical learning share general mechanisms of implicit regularity detection, then statistical learning performance in a non-auditory modality using non-linguistic stimuli should predict individuals' perceptual learning for speech comprehension.

Perceptual learning in speech and statistical learning may also draw (partly) on the same underlying cognitive abilities, such as working memory and attention. Therefore, we investigated whether both types of learning could be predicted from general cognitive and linguistic abilities. Ahissar and Hochstein (2004) proposed that attentional mechanisms may be engaged in choosing which neuronal populations pass on task-relevant information to the higher levels and in increasing the functional weights of these populations. Several frameworks of perceptual learning incorporate the idea that attentional mechanisms are involved in perceptual learning (e.g., Goldstone, 1998; Fahle, 2006; Dosher et al., 2010). A study on frequency discrimination found that perceptual learning even occurred after training with non-discriminable stimuli (Amitay et al., 2006). Apparently, training directed the participants' attentional focus to the relevant stimulus dimension, which was sufficient to access the relevant low-level representations during the test phase (Amitay et al., 2006). Moreover, performance on a selective attention task predicted the amount of learning in adaptation to accented speech (Janse and Adank, 2012). Further evidence that attention is involved in perceptual learning comes from studies in which listeners were simultaneously exposed to noise-vocoded speech and both auditory and visual distractors (Huyck and Johnsrude, 2012; Wild et al., 2012). Only listeners who attended the noise-vocoded stimuli showed improved performance in recognizing noise-vocoded speech. Similar effects of attentional focus arise in tasks of visual statistical learning. When observers are asked to attend to symbols of a certain color in a two-color symbol stream, statistical learning effects unfold for regularities within the attended color but not for regularities within the unattended color (Turk-Browne et al., 2005). These findings imply that only attended features are effectively learned. It has been proposed that training procedures that facilitate participants to *switch* their attention to appropriate perceptual features (e.g., fixed temporal presentation of multiple stimuli, repeated presentation) may particularly enhance perceptual learning (Zhang et al., 2008). Therefore, attention switching control may be involved in the process of distinguishing relevant from non-relevant features in tasks of implicit learning.

Another cognitive ability that may be involved in tasks of implicit learning is working memory, which is required to simultaneously store and process auditory or visual information (Gathercole, 1999). Performance on working memory tasks has been shown to predict performance in various speech reception tasks (for a review see Akeroyd, 2008) and, more specifically, there are indications that working memory relates to perceptual learning performance. Teenaged students with learning and reading disabilities who participated in perceptual learning tasks of frequency and duration discrimination showed improved working memory skills after training (Banai and Ahissar, 2009). Furthermore, the two students who failed to show perceptual learning were characterized by the poorest working memory capacity in the sample. During training, students were repeatedly presented with the same stimuli, which allowed them to access low-level representations, thereby improving frequency and duration discrimination. Thus, working memory may have aided perceptual learning by keeping stimuli accessible (also see Goldstone, 1998). In contrast to these findings, Erb et al. (2012) did not find an association between working memory and individual differences in adaptation to noise-vocoded speech. Note, however, in this study, working memory was measured by tasks that relied on immediate recall and, hence, on short term memory (i.e., non-word repetition task, digit span forward task). Possibly, more complex span tasks, that measure the ability to simultaneously store and process information, rather than just recall capacity, may be particularly associated with tasks of perceptual learning. With respect to statistical learning, recent studies reported correlations between working memory capacity and performance on implicit sequence learning tasks (Bo et al., 2011, 2012). However, findings regarding the link between working memory and implicit learning of sequences are controversial (for a review see Janacsek and Nemeth, 2013) and it has been argued that working memory as an executive resource is not involved in tasks of implicit learning (Kaufman et al., 2010).

An additional cognitive ability that should be considered is processing speed. Processing speed reflects the efficiency of a processing system to perform simple operations (Kaufman et al., 2010) and as a general index of processing efficiency, may be assumed to facilitate perceptual learning. Previous research showed that processing speed correlates with performance on tasks of implicit sequence learning (Salthouse et al., 1999; Kaufman et al., 2010). Higher efficiency of the processing system may be beneficial at various stages of the adaptation process. In the framework of the RHT, processing speed may reduce listeners' time to retrieve high-level representations and to initiate modification processes. Furthermore, processing speed may accelerate the process of weight retuning, thereby gaining faster access to low-level representations.

As the current study focuses on adaptation for spoken language understanding, perceptual learning may also draw on linguistic knowledge. Davis et al. (2005) presented data on how the so-called pop-out effect accelerates the process of perceptual learning: if listeners knew the content of what was going to be said before they actually heard the sentence in its degraded form, this benefited their perceptual learning. In line with the Eureka effect in the RHT, in which a cue regarding the content of the stimulus can trigger direct perception of the stimulus and facilitates strong and long-lasting learning effects (Ahissar and Hochstein, 2004), this pop-out finding suggests that lexical knowledge facilitates access to higher-level representations, thereby initiating top–down processes that aid sublexical retuning (Davis et al., 2005). Accordingly, vocabulary, as a measure of lexical knowledge, has been found to predict the amount of perceptual learning in listeners who were adapting to an unfamiliar foreign-sounding accent (Janse and Adank, 2012), accents being linguistic degradations of the stimulus. If we assume that lexical knowledge aids perceptual learning by guiding the top–down search, effects of lexical knowledge should also arise in non-linguistic speech degradations. Therefore, we investigate whether linguistic knowledge, as indexed by vocabulary knowledge, may also facilitate shifting of attention to relevant features of acoustically degraded speech.

As we want to investigate which cognitive processes are involved in perceptual learning in speech, we also aim to test whether our findings generalize to a heterogeneous group of listeners. Older adults typically form a highly heterogeneous group, as perceptual and cognitive processing undergo changes over the life span. Age-related changes in hearing acuity (Lin et al., 2011), processing speed, capacity on working memory tests, attentional control (for a review see Park and Reuter-Lorenz, 2009) but also lexical knowledge (Ramscar et al., 2014) may therefore help to identify relevant cognitive processes. Importantly, the ability to adapt to unfamiliar speech input is preserved throughout the life span (Peelle and Wingfield, 2005; Golomb et al., 2007; Adank and Janse, 2010; Gordon-Salant et al., 2010). However, differences in the amount and pattern of perceptual learning over exposure between younger and older adults also indicate changes in the underlying processes. While younger and older listeners show the same amount of learning in the initial adaptation phase, older listeners' performance plateaus earlier in adapting to unfamiliar speech (Peelle and Wingfield, 2005; Adank and Janse, 2010), older adults show less transfer of learning to similar conditions (Peelle and Wingfield, 2005), and exhibit slower consolidation of learning (Sabin et al., 2013). Such differences illustrate that the interdependency between cognitive functions and implicit learning processes may change as a function of age. Cognitive abilities associated with adaptation to unfamiliar speech in younger adults may not be the same as in older adults. In order to gain more insights into individual abilities associated with adaptation to unfamiliar speech across the life span, we tested both younger and older adults.

In sum, this study investigates perceptual learning for spoken language understanding in younger and older adults. We use noise-vocoded speech, an acoustic degradation of the speech signal which simulates the auditory signal of a cochlear implant. In contrast to naturally occurring variability in speech (such as accents), participants do not encounter noise-vocoded speech in everyday life. As a consequence, all participants share the same naïve exposure level. We specifically study whether perceptual learning is associated with a general ability to implicitly detect statistical regularities. By testing participants' probabilistic sequence learning with visual non-linguistic stimuli, we apply a rigorous test of the association between the two types of implicit learning. Additionally, we investigate whether both types of implicit learning are associated with individual differences in attention switching control, working memory, information processing speed or lexical knowledge.

## Materials and methods

### Participants

In total, 60 younger and 73 older adults participated in the current study. All participants were native speakers of Dutch, neurologically intact and had no history of language disorders. One younger participant was excluded as he showed floor performance throughout the perceptual learning task (i.e., he did not understand the noise-vocoded speech at all). Younger adults were aged between 18 and 29 years (mean age 21 years, *SD* 2.5 years) and older adults were aged between 60 and 84 years (mean age 68.4 years, *SD* 5.7 years). In both age groups, the majority of participants were female (53 out of 59 participants in the younger and 47 out of 73 participants in the older sample). Participants had normal or corrected-to-normal vision. Participants were recruited via the subject database of the Max Planck Institute for Psycholinguistics and were compensated € 8 per hour for their time.

### Auditory, cognitive, and linguistic background measures

#### Auditory measure

**Figure 1:**
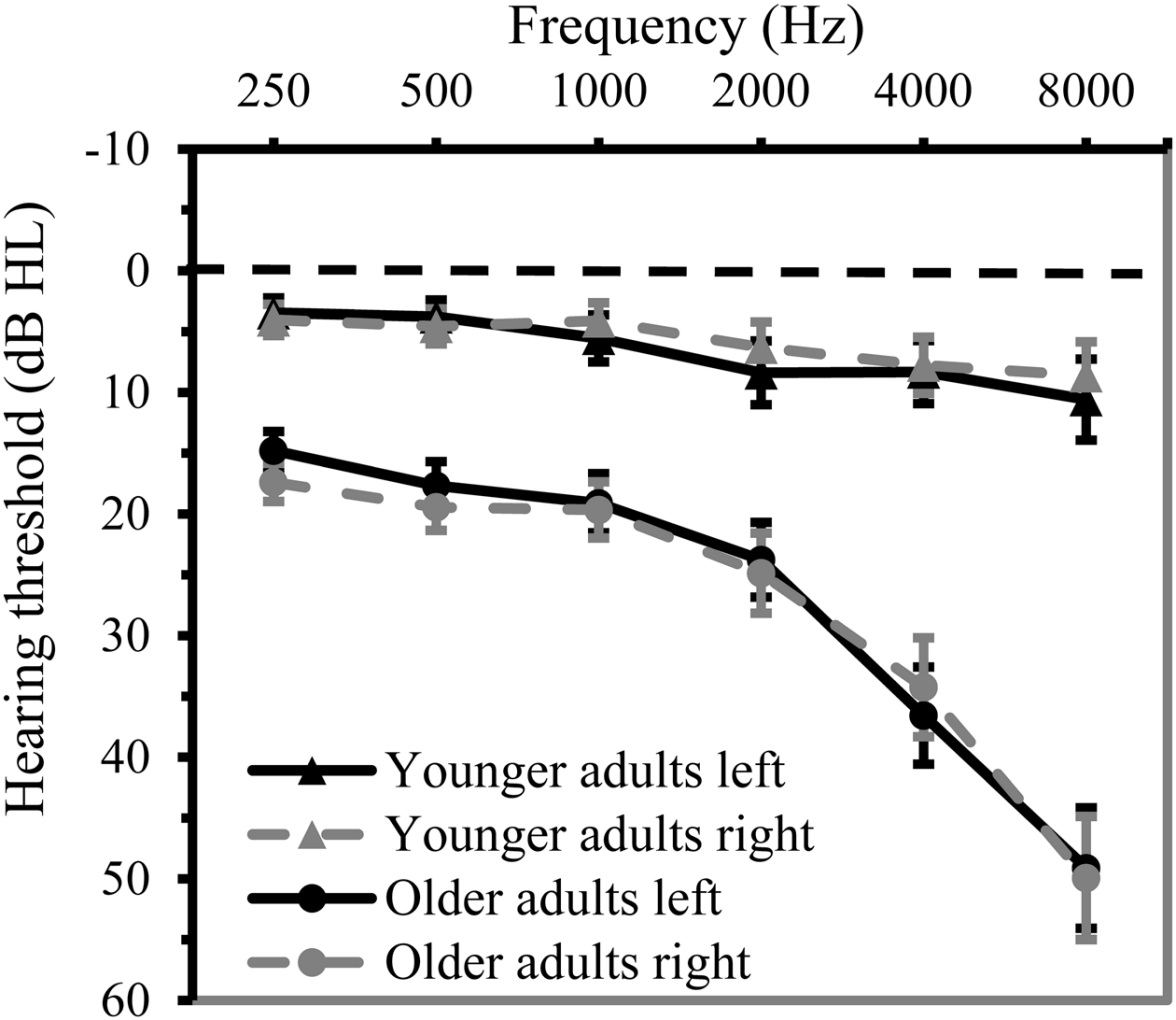
**Mean hearing threshold (in dB HL) at 0.25, 0.5, 1, 2, 4, and 8 kHz for both ears in younger (*n* = 59) and older adults (*n* = 73)**. Error bars indicate two standard error from the mean.

***Hearing thresholds***. Age-related hearing loss is prevalent in older adults (Lin et al., 2011). Poorer hearing may affect perceptual learning as auditory input contains less detail, thereby interfering with accessing and retuning low-level representations. Participants' auditory function was assessed by measuring air-conduction pure tone thresholds with the aid of an Oscilla USB-300 screening audiometer. As age-related hearing loss particularly affects sensitivity to high frequencies, a high-frequency pure tone average [PTAH] was taken as index of hearing acuity. This PTAH was calculated as the mean hearing threshold over 1, 2, and 4 kHz (instead of the standard PTA over 0.5, 1, and 2 kHz). Only the PTAH of the best ear was entered in the analysis, as all auditory stimuli were presented binaurally. Twenty-seven older participants actually qualified for hearing aids on the basis of their hearing thresholds according to the standard of hearing-aid coverage in the Netherlands (PTAH of the worst ear ≥35 dB HL). None of the participants wore hearing aids in daily life, however. Higher thresholds reflected poorer hearing. Mean thresholds at different frequencies per age group are given in Figure 1.

#### Cognitive measures

***Working memory***. Participants performed a digit span backward task as an index of working memory capacity. The test was a computerized variant of the digit span backward task included in the Wechsler Adult Intelligence Scale Test (Wechsler, 2004) and presented via E-prime 1.2 (Schneider et al., 2002). Participants were asked to report back sequences of digits in reverse order. Digits were presented in a large white font (Arial, font size 100) against a black background. Each digit was presented for 1 s with an interval of 1 s between the consecutive digits of a sequence. Sequence length increased stepwise from two to seven digits and performance on each sequence length was tested on two different trials (all participants were presented with all sequence lengths, regardless of their performance on earlier easier trials). The actual test trials were preceded by two practice trials with a sequence length of three to familiarize participants with the task. Participants had to recall 12 test sequences in total. Individual performance was operationalized as the proportion of correctly reported sequences (out of 12).

***Processing speed***. Information processing speed was assessed by means of a digit symbol substitution task. Participants had to convert as many digits as possible into assigned symbols in a fixed amount of time (90 s). The digit symbol substitution task is a paper-and-pencil test that was derived from the Wechsler Adult Intelligence Scale Test (Wechsler, 2004). Performance was measured by the number of correctly converted digits in 90 s, meaning that higher scores reflected higher information processing speed.

***Attention switching control***. The Trail Making Test was administered to obtain a measure of attention switching control. The paper-and-pencil test contained two parts. In Part A, participants were asked to connect numbers as quickly as possible in ascending order (i.e., 1-2-3…), the numbers being spread randomly over a white page. The Part B page had both numbers and letters randomly spread over the page. Participants now had to alternately join numbers and letters in ascending order (i.e., 1-A-2-B-3-C…). In both parts, 25 items had to be connected and the total time to complete each part was measured. We calculated the ratio between both parts (Part B/Part A) as measure of attention switching control (Arbuthnott and Frank, 2000), thereby taking general slowing into account (Verhaeghen and De Meersman, 1998; Salthouse, 2011). Higher scores indicated higher costs of switching between letters and numbers, therefore, poorer attention switching control.

#### Linguistic measure

***Vocabulary knowledge***. A vocabulary test in the form of multiple choice questions was administered to obtain a measure of linguistic knowledge (Andringa et al., 2012). The computerized test was administered in Excel (Courier font size 15). Participants had to indicate which out of five possible answers was the correct meaning of Dutch low-frequency words, the last alternative always being “I don't know.” Words were not domain-specific and each target word was embedded in a different, neutral carrier phrase. The vocabulary test consisted of 60 items. There was no time limit or pressure to complete the test. Performance was measured by test accuracy, that is, the proportion of correct answers (out of 60). Higher scores thus reflected greater vocabulary knowledge.

### Statistical learning

#### Materials and design

To investigate statistical learning, we adopted the artificial grammar learning—serial reaction time (RT) paradigm (Misyak et al., 2010a). This paradigm has typically been used in studies on statistical learning in language processing and has been found to link to individual language processing abilities (Misyak et al., 2010a,b; Misyak and Christiansen, 2012). As artificial grammar learning simulates language learning processes, the task makes use of auditory presented sound sequences such as non-words. However, as we wanted to investigate whether individuals' ability to adapt to an unfamiliar speech condition could be predicted by a general ability to implicitly detect regularities, we used visual and non-linguistic stimuli in the statistical learning task. That is, we applied a rigorous test for the relationship between statistical learning and perceptual learning by preventing that a relationship between both measures of learning was specific for auditory and linguistic processing.

**Figure 2:**
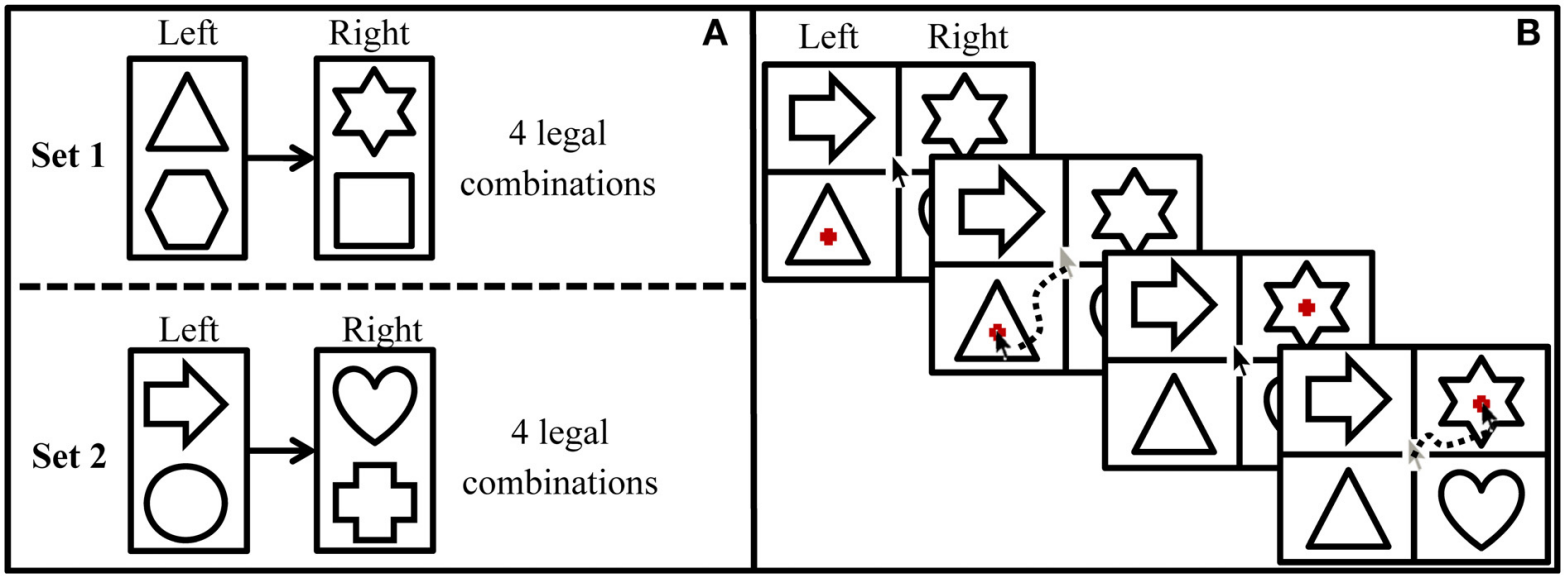
**Structure of the statistical learning task. (A)** Structure of the grammar in which the first target is always displayed on the left side of the screen and the second target is always displayed on the right side of the screen. **(B)** Procedure of a grammatical trial during the exposure phase.

Participants were presented with familiar, geometrical shapes in a 2 × 2 design on the computer screen (see Figure 2B), in which one shape on either side of the screen served as target and one as distractor item. Target shapes were sequentially highlighted by a visual marker and participants' task was to click as fast as possible on the highlighted target. The first target was always one on the left side of the screen (i.e., upper or lower one in the first column) and the second target was always on the right side of the screen (i.e., upper or lower one in the second column). The second target was only highlighted after the participant had clicked on the first target item. Crucially, which of the two items in the right-hand column would be highlighted was predictable on the basis of the first target [e.g., in Figure 2B, a *triangle* would always be followed by a *star* or a *square* (*the latter is not in the display*), but never by a *heart*].

Materials consisted of eight familiar, geometrical shapes drawn with a single, continuous black line. The shapes were divided into two grammatical subsets of four shapes each (i.e., Set 1: *triangle, hexagon, star, square*; Set 2: *arrow, circle, heart, cross*). Within each set, two items were selected to appear as first targets (i.e., Set 1: *triangle, hexagon*; Set 2: *arrow, circle*) and were always followed by one of the other two items that served as second targets (i.e., Set 1: *star, square*; Set 2: *heart, cross*). Therefore, four combinations of shapes were grammatical within each set, resulting in a total set of eight grammatical combinations (see Figure 2A). Target items were presented along with distractors in a rectangular grid display on the computer screen (see Figure 2B). Distractor items were shapes from the subset that was currently not tested and the two distractor shapes on the screen formed a grammatical combination themselves. Thus, within a grammatical trial, the transitional probability from the first to the second target was 1, as the first target could only be followed by the target from the same subset. Within the grammar, however, the transitional probability between two adjacent items was 0.5, as a target was followed by a specific successor only half of the time (i.e., a *circle* being followed by either a *heart* or a *cross*, see Figure 2A). Target positions were randomly assigned such that it was unpredictable whether a first or second target would be displayed in the upper or lower row of a particular column.

The artificial grammar learning task was composed of blocks and split into an exposure phase, a test phase and a recovery phase. During the exposure phase, participants could learn the grammar by picking up on the co-occurrence probabilities of the shapes. In total, the exposure phase consisted of 16 grammatical blocks. Within each block, all grammatical combinations were repeated once, resulting in 128 exposure trials (8 × 16). The test phase consisted of two ungrammatical blocks (2 × 8 trials). In these ungrammatical blocks, the original grammar was reversed, such that a target was followed by targets of the other (competing) subset. Participants who implicitly learned the grammar should show a drop in performance as they would need to correct their predictions, resulting in a slowed response to the second target. This measure of learning is widely accepted in the literature on implicit learning (Janacsek and Nemeth, 2013): a drop in performance due to removing the underlying regularities can only be linked to grammar sensitivity, whereas learning measures in terms of improvement during the exposure phase cannot be teased apart into general task learning and statistical learning. Therefore, statistical learning was operationalized by the difference in task performance between the last four blocks of the exposure phase (blocks 13–16) and the subsequent ungrammatical test phase (blocks 17–18). The recovery phase again consisted of two grammatical blocks and serves as a control phase. If participants learned the grammar, by re-introducing the regularities in the recovery phase, participants' performance should not decrease any further. In total, the artificial grammar learning task thus contained 20 blocks and 160 trials (8 × 20).

#### Procedure

The artificial grammar learning task was presented in E-prime (Schneider et al., 2002) and started with five practice trials that were all grammatical. Participants were instructed to click as quickly as possible on target shapes that were marked by a small filled red cross (10 × 10 mm) in the center of the target shape. Participants were informed that they had to click on two successive targets and that the first target would be located in the first column and the second target would be located in the second column. Each trial started with the presentation of the visual display that consisted of the four shapes and two grid lines, marking the four quadrants on the screen. At the start of each trial, the mouse cursor was located in the center of the screen. Each shape was displayed in a size of 75 × 70 mm. The visual marker appeared in the middle of the first target shape 500 ms after the onset of the visual display, and was shown until the participant clicked on the marked picture. After the participant had responded, the mouse cursor was automatically set back to the center of the screen to ensure the same distance for all click responses. The second visual marker (same red cross now marking the second target shape) appeared 500 ms after the first click. This time interval was implemented in the design to allow for prediction effects, even in the adults who had slower processing. This time interval had been successfully applied in an earlier study on implicit sequence learning in older adults (Salthouse et al., 1999). Participants could not make errors: the experiment only proceeded if a participant clicked on the appropriate target shape. Clicking on a distractor shape or outside the target picture before giving a correct click resulted in a higher RT. The intertrial-interval was 500 ms. After each block, a small break of 2500 ms was implemented to avoid fatigue effects. During this break, participants saw the block number of the upcoming block and a reminder to click as quickly as possible. It took approximately 20 min to complete the task.

To assess statistical learning, we measured latencies from target highlighting to the subsequent mouse response. Facilitation scores were calculated to index individuals' sensitivity to implicit regularities. The facilitation score was calculated by dividing the RT to the first, unpredictable target within a trial by the RT to the second, predictable target within the same trial. Thus, RT to the first target served as baseline performance within each trial. This was important to minimize biases of task learning and motor performance, particularly for those older adults who may have had little practice in using a computer mouse. During the course of the experiment, RTs may generally get faster as older adults get more experienced in using a mouse. By implementing a new baseline within each new trial, such motor learning should be accounted for. If participants cannot predict which target will be highlighted next, their RTs to both targets within a trial will be similar and will result in a facilitation score of 1. During the exposure phase, learning manifests itself in an increasing facilitation score. That is, if participants learn to predict the second target, RTs to the second item will be faster and, therefore, shorter compared to the first, unpredictable target RTs.

### Perceptual learning

#### Materials and design

Sixty Dutch sentences were noise-vocoded to create an unfamiliar speech condition to which participants needed to adapt. In noise-vocoded speech, frequency information in the signal is replaced by noise while preserving the original amplitude structure over time. The speech signal was split into multiple non-overlapping frequency bands, which approximately matched equal distances on the basilar membrane (Greenwood, 1990). From each frequency band the smoothed amplitude envelope was derived and imposed on wide-band noise in the same frequency range. In a last step, these modulated noise bands were recombined, creating a speech signal that sounded like a harsh robot voice. All signal editing was done in Praat (Boersma and Weenink, 2011).

An important characteristic of noise-vocoded speech is that the comprehension level of the speech signal can easily be manipulated by varying the number of frequency bands. The more frequency bands are used to decompose the speech signal, the more detail of the original temporal and amplitude structure is preserved and the more intelligible the speech signal is. Previous research has shown that 10 frequency bands are enough for naïve listeners to immediately understand more than 90% of noise-vocoded speech (Sheldon et al., 2008). However, when presented with speech noise-vocoded with fewer bands, participants only reach this level of performance after a certain amount of exposure.

The maximal amount of learning or intelligibility improvement can be observed if the starting level is neither too high nor too low, so that sufficient information can be derived from the acoustic materials to initiate learning while at the same time allowing for sizeable improvement (see Peelle and Wingfield, 2005). We initially tried to provide participants with an individual starting level from which they could still show improvement. In a separate pilot study, we therefore assigned 23 older adults to a specific noise-vocoding condition (i.e., 4 or 6 bands) on the basis of their performance on a speech reception threshold (SRT) task in noise. Inspection of the data showed that participants' starting level clustered according to band condition. Older adults in the 4 band condition showed a very low starting level (on average they understood only 10% of the sentences correctly), whereas older adults in the 6 band condition showed a very high starting level (on average they already understood 65% of the sentences). Relatedly, the correlation between SRT result and initial performance on the noise-vocoded speech was weak. As our attempt to individualize starting levels on the basis of a speech-in-noise task was not successful, we aimed to provide a roughly similar starting level for both age groups. Based on the results of the pilot study, we decided to present older adults with speech that was vocoded with 5 bands (corner values using 5 frequency bands: 50-280-757-1742-3781-8000 Hz). As younger adults understand more when being exposed to the same degradation as older adults (Peelle and Wingfield, 2005; Sheldon et al., 2008), we presented younger adults with four-band speech (corner values using 4 frequency bands: 50-369-1161-3125-8000 Hz), thus, a more difficult speech condition than older adults (cf. Golomb et al., 2007). Consequently, we were able to see sizeable and comparable amounts of improvement over the course of exposure in both age groups.

Sentences were selected from audiological test materials (Versfeld et al., 2000) and were all produced by the same, male speaker. Each sentence had a length of eight or nine syllables and contained four keywords. Keywords in the selected set of sentences included a noun, verb and preposition. The fourth keyword was an adjective, adverb or a second noun. An example sentence “*De sneeuw glinstert in het maanlicht*” (“*The snow is glistening in the moonlight*”) contained the keywords “*sneeuw*,” “*glinstert*,” “*in*,” and “*maanlicht*.” Note that five additional sentences were selected for practice purposes, so that there was no overlap in sentence content between practice and test items. Practice sentences had the same length as test items (a list of all sentences used in the current study is provided in Supplementary Material).

#### Procedure

An auditory sentence identification task was administered to investigate perceptual learning using the experiment program E-prime (Schneider et al., 2002). Participants listened to the noise-vocoded sentences and were asked to identify and repeat these sentences. They were encouraged to guess if they were unsure. Participants were first presented with five practice trials. First, participants listened to three clear sentences to familiarize them with the task and the speaker. Moreover, these practice trials were used to check whether participants' memory span was sufficient to perform the task given clear input, which was the case for all participants. Then participants listened to two sentences that were noise-vocoded with only two frequency bands to present them with the type of degradation. This more difficult condition with fewer bands was chosen to make sure that no learning could occur during the practice phase (e.g., Ahissar and Hochstein, 1997; Pavlovskaya and Hochstein, 2004; Liu et al., 2008). Practice trials were identical for all participants and were presented in the same order. In contrast, the 60 test sentences were presented in random order for each participant, so that observed learning effects would be independent of inherent intelligibility differences between sentences (e.g., due to differences in semantic predictability). Participants heard a short (125 ms) 3.5 kHz tone to call their attention to the upcoming stimulus 500 ms before sentence onset. After each sentence, the researcher scored the number of correctly repeated keywords (0–4) online. The next trial started immediately after the researcher had confirmed the scoring of the previous trial. Auditory stimuli were presented binaurally via dynamic closed, circumaural headphones (Sennheiser HD 215), at a level of 85 dB SPL. Participants' answers were audiorecorded to allow for later checking of their responses.

### Experimental procedure

Measures of younger adults were obtained in a single experimental session. Testing was spread over two sessions for the older adults, as they also participated in a different study. During the first session, older adults performed the background measures described above. The second session consisted of the statistical learning and the perceptual learning task and followed within a month on the first session. In both age groups, tasks were presented in a fixed order. Although the order differed between younger and older adults, the statistical learning task was always presented before the perceptual learning task. All participants were tested individually in a sound-attenuating booth to minimize distraction. Before the start of each task, participants received verbal and printed task instructions. Participants could ask questions at any time. Between tasks, participants were encouraged to take small breaks.

### Data analysis

#### Statistical modeling

To assess learning performance, we implemented linear mixed-effects models using the lmer function from the lme4 package (Bates et al., 2012) in R (version 2.15.1). In this way, both participants and items could be assessed as random factors and the maximal random slope structure of models could be defined to reduce the probability of a type 1 error (Barr et al., 2013). First, we modeled statistical and perceptual learning performance as a function of age group to assess whether younger and older adults differed in their learning performance. Second, we analyzed the contributions of individual abilities in learning separately within each group as our focus was on individual differences within the respective age groups. Thus, the modeling process that is described here was applied to the statistical learning data and to the perceptual learning data of both age groups.

Linear regression models are based on the assumption that the predictors included in the analysis do not show collinearity (Baayen, 2012). Although some predictor measures were intercorrelated (see Section Performance on Background Measures), we did not control for these intercorrelations for two reasons. First, most correlations explained less than 20% of the variance in the correlated measure (i.e., with correlation coefficients below 0.45). Only the correlation between age and speed in the older adults was moderately correlated (*r* = −0.562). Second, simultaneous inclusion of correlated measures in the analysis has been shown to provide a more reliable interpretation of estimates than inclusion of residualized variables (York, 2012; Wurm and Fisicaro, 2014).

Statistical learning was defined as a drop in performance in the test phase (blocks 17–18) compared to the performance at the end of the exposure phase (blocks 13–16). Therefore, in models of statistical learning, the fixed categorical variable phase (exposure vs. test phase) was the variable of interest to predict individuals' facilitation scores and to indicate learning. Additionally, two control variables and the corresponding two- and three-way interactions with phase were included in models of statistical learning. Control variables were the categorical variable “first target position” (was the first target displayed in the upper or lower row of the left column?) and the categorical variable “target alignment” (were the two targets in a trial aligned horizontally or diagonally?). Given the directionality of Western writing systems, we expected a first target position effect as participants may click faster on a target in the upper left quadrant than in the lower left quadrant. We also expected the drop in facilitation score during the test phase to be less distinct in trials with the first target appearing in the upper left quadrant, such that target position was expected to interact with the amount of learning. Moreover, the alignment of targets was thought to affect second target RTs. Note that the experimental program always set the mouse back to the center of the screen after each click. Despite this automatic mouse reset, participants tended to also move the mouse back to the center of the screen. By doing that, participants unintentionally initiated a movement toward the diagonal shape. Therefore, we assumed that participants would be faster in responding to the second target if targets were arranged diagonally rather than horizontally (see Figure 2), which would result in higher facilitation scores. This direction effect may interact with the effect of removing the regularities, such that the grammaticality effect be decreased for the diagonal movements.

In models of perceptual learning, the number of correctly repeated keywords per sentence served as index of recognition performance and was entered as numerical dependent variable into the model. As perceptual learning was defined as the improvement in speech understanding over exposure, we split the experiment into six blocks, containing 10 sentences each and added block as numerical measure of exposure to the model. However, before block was included in the analysis, we performed a log-transformation of block, as perceptual learning has typically been described by fast initial learning that levels off with increasing exposure (see also **Figure 4**). The transformation of block therefore provided us with an index of exposure that took this non-linear improvement curve into account and converted the improvement over exposure into a linear scale^1^.

In the first step of the analysis, we identified the maximal random slope structure of our models to allow for the fact that different participants or items may vary with regard to how sensitive they are with respect to the variables at hand (Cunnings, 2012; Barr et al., 2013): if, e.g., vocabulary knowledge only matters for the understanding of some sentences in the perceptual learning task but not for others, the effect of vocabulary should be modeled individually for each sentence and removed from the fixed effect structure. Changes in the random-slope structure were evaluated by means of the Akaike information criterion (AIC). The model with the lower AIC value (difference ≥2) and, therefore, better model fit was retained. As we were interested in the predictors of individual amount of learning, a random participant slope of phase was included in all models of statistical learning. Accordingly, in models of perceptual learning, a random participant slope of block was inserted. That is, all models calculated the learning effect (i.e., the effect of phase in statistical learning and the effect of block in perceptual learning, respectively) individually for each participant.

After determining the maximal random slope structure, we first performed an age group comparison by testing the interactions between age group and the respective index of learning (i.e., phase or block). As younger and older adults may differ with respect to the effects of target position and target alignment on their learning performance, all possible two-way interactions between grammaticality, age group, target alignment and first target position and the three-way interactions between (1) age group, grammaticality and target position and between (2) age group, grammaticality and target alignment were included in the age group comparison of statistical learning.

In a second step, we assessed which cognitive abilities may facilitate learning within the separate age groups. In the statistical learning analysis, the best model that explained the facilitation score on basis of the interactions between phase, target position and target alignment was taken as initial model. In the perceptual learning analysis, the initial model only contained block. Then, measures of age (in older adults only), hearing sensitivity (in models of perceptual learning only), statistical learning performance (in models of perceptual learning only), attention switching control, working memory, processing speed and vocabulary (all evaluated as numerical covariates) and their interaction with phase (in models of statistical learning) or with block (in models of perceptual learning) were added simultaneously to the initial model. This method of forced entry was preferred, as we had no prior theoretical assumptions about the relative importance of each predictor and aimed to identify those predictors that had unique exploratory power in predicting facilitation scores. All individual predictor measures were centered around their mean prior to inclusion. After we had entered the individual predictor measures, we adopted a backward stepwise selection procedure, in which first interactions and then predictors were removed if they did not attain significance at the 5% level. Each change in the fixed effect structure was evaluated in terms of loss of model fit by means of a likelihood ratio test. Results of the analysis are indicated in estimated absolute effect sizes (β), standard errors, *t*-values and *p*-values. Note however that the current version of the lme4 package does not report *p*-values for *t*-tests in models with a maximal random slope structure, as it is presently unclear how to calculate the appropriate number of degrees of freedoms (Baayen, 2012). Reported *p*-values were, therefore, derived by performing a likelihood ratio test between a model that included the specific fixed effect or interaction and a model that did not while all other model parameters were kept constant. That is, *p*-values actually reflect the significance of loss in model fit if the effect or interaction was excluded from the model.

#### Individual measure of statistical learning performance

As we wanted to assess whether individual statistical learning performance predicts adaptation to noise-vocoded speech, we needed an index of statistical learning ability for each participant. We derived this index by calculating the random participant slopes of phase (individual adjustments to the general slope) on the basis of the most parsimonious model, in which facilitation scores were predicted only by phase and the control variables (i.e., we derived the measure of statistical learning ability before we included effects of individual predictor measures in the above mentioned analysis).

Thus, we determined an individual value for each participant with which the general effect of phase (in the fixed structure of the model) had to be adjusted to resemble his/her individual learning effect. The lower the value, the more negative was a participant's slope when changing from the end of the exposure phase to the test phase, indicating a steeper drop in facilitation score and, hence, more statistical learning.

## Results

### Performance on background measures

Mean performance of younger and older adults and age group differences on all background measures are displayed in Table 1. As expected, hearing acuity was better in younger adults (i.e., thresholds were lower) than in older adults. Moreover, younger adults showed faster processing and larger memory capacity than older adults. On average, older adults were able to correctly repeat 5.62 test sequences in the working memory test, which corresponds to a mean digit span of four. Younger adults correctly repeated 8.08 test sequences, corresponding to a mean digit span of five. No difference could be observed in attention switching control between age groups. Older adults outperformed younger adults on the vocabulary test. However, older adults also showed relatively little variation on the vocabulary test [coefficient of variation (*SD/M*) = 6.9%]. Statistical testing confirmed that the variance in older adults' vocabulary scores was significantly lower than the variability in younger adults' data (coefficient of variation = 11.8%) (Levene's Test: *F* = 4.15, *df*_1_ = 1, *df*_2_ = 130, *p* = 0.044).

**Table 1:** **Mean performance per age group and age group differences on cognitive, linguistic, and auditory measures**.

**Measure**	**Younger adults**		**Older adults**		**Age group difference**	
	***M***	***SD***	***M***	***SD***	***t***	***p***
Working memory	67.37	17.18	46.80	18.72	6.57	<0.001
Processing speed	68.10	9.44	48.73	11.01	10.88	<0.001
Vocabulary	0.68	0.08	0.87	0.06	−15.80	<0.001
Attention	1.97	0.44	2.05	0.62	−0.96	0.340
Hearing (PTAH)	0.90	5.56	23.31	10.28	−15.96	<0.001

*t-tests tested two-tailed*.

Intercorrelations between background measures within each age group are reported in Table 2. In younger adults, significant correlations were observed between the cognitive measures of working memory and processing speed and between working memory and vocabulary. The same intercorrelations were also observed in the older adults. As expected, age correlated with hearing sensitivity and with processing speed within the older sample: older–older participants generally had poorer hearing and slower processing than younger–older participants. Moreover, processing speed was related to hearing sensitivity in older adults. However, when both measures (i.e., speed and hearing) were controlled for age, this correlation was no longer significant (*r* = −0.128, *p* = 0.279, *df* = 71).

**Table 2:** **Pearson's correlation coefficients between measures of cognitive, linguistic, and auditory functioning per age group**.

**Measure**	**Attention**	**Memory**	**Speed**	**Vocabulary**	**Hearing**
**YOUNGER ADULTS (*n* = 59)**					
Attention switching	1				
Working memory	−0.109	1			
Processing speed	0.079	0.301^*^	1		
Vocabulary	−0.071	0.311^*^	−0.156	1	
Hearing	−0.097	−0.233	0.022	−0.119	1
**OLDER ADULTS (*n* = 73)**					
Attention switching	1				
Working memory	−0.211	1			
Processing speed	−0.187	0.311^**^	1		
Vocabulary	−0.076	0.250^*^	0.207	1	
Hearing	0.104	−0.170	−0.336^**^	−0.113	1
Age	0.059	−0.194	−0.562^**^	0.017	0.426^**^

* *p < 0.05*,

** *p < 0.01 (tested two-tailed)*.

### Statistical learning

**Figure 3:**
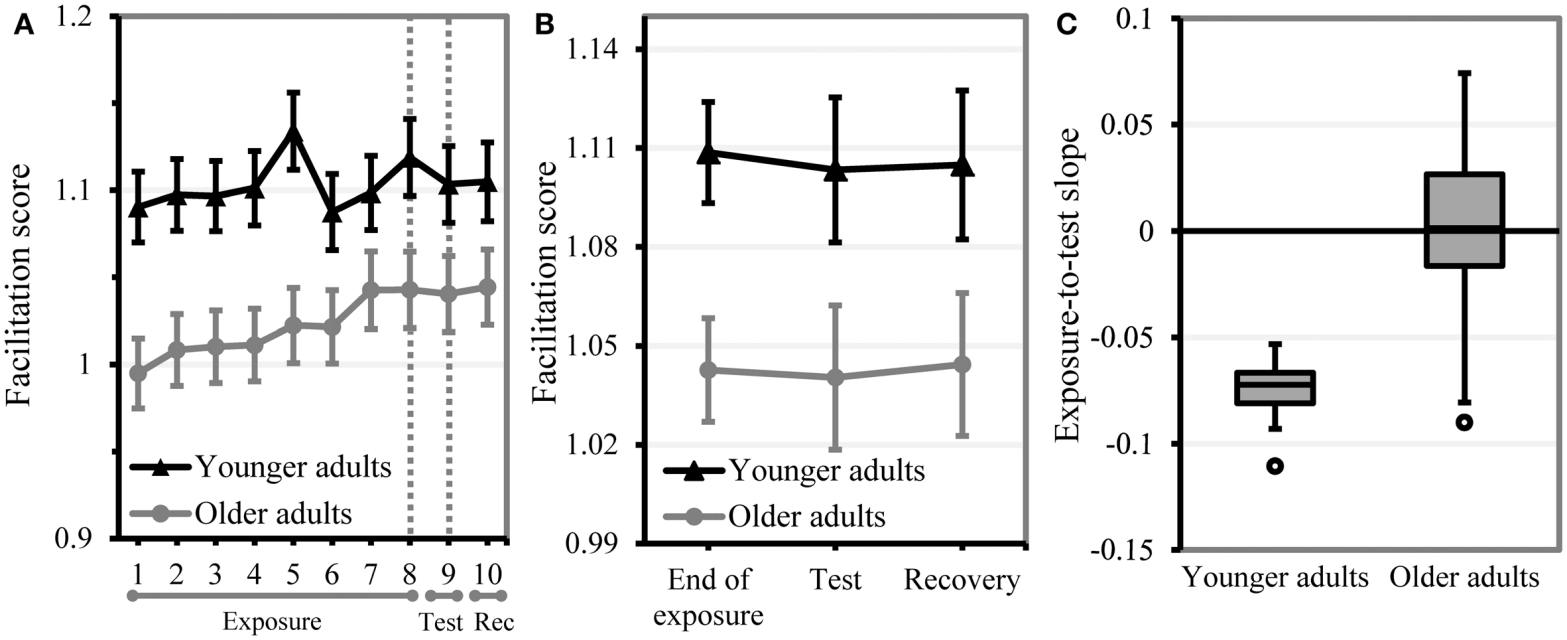
**Performance on the statistical learning task**. A drop in facilitation score from the end of the exposure phase (blocks 7–8) to the test phase (block 9) indicates learning. Error bars indicate two standard errors from the mean. **(A)** Mean statistical learning performance per age group and block. The area between the dotted lines represents where the effect of removing the underlying regularities should be observed. **(B)** Mean statistical learning performance per age group and phase. **(C)** Boxplot of statistical learning performance in younger and older adults (individual exposure-to-test slopes from the statistical model). More negative slopes reflect more learning.

Valid facilitation scores were restricted to those within 2.5 *SD* from the mean facilitation score within each age group. Table 3 shows the average performance of younger and older adults on the statistical learning task in terms of response times and facilitation score. As expected, younger adults were significantly faster in responding to the first target (*t* = −84.30, *df* = 23249.45, *p* < 0.001) and to the second target (*t* = −104.34, *df* = 23585.75, *p* < 0.001) than older adults. Note that all responses in the statistical learning task were accurate as the experimental task only proceeded when a participant had clicked on the correct shape. Figure 3A shows the average facilitation scores for both age groups over block^2^. Figure 3B displays the mean facilitation scores at the end of the exposure phase, in the test phase and in the recovery phase to illustrate the learning effect. Moreover, the range of statistical learning that was observed within each age group is displayed in Figure 3C. Estimates of the best model within each age group are displayed in Table 4.

**Table 3:** **Mean response times (in ms) and facilitation scores of younger adults (*n* = 59) and older adults (*n* = 73) on the statistical learning task**.

	**1st target RT**		**2nd target RT**		**Facilitation score**	
	***M***	***SD***	***M***	***SD***	***M***	***SD***
Younger adults	499	130	473	129	1.103	0.328
Older adults	705	243	728	240	1.024	0.359

**Table 4:** **Statistical models for the facilitation score of younger and older adults in the statistical learning task**.

**Fixed effects**	**Younger adults**					**Fixed effects**	**Older adults**				
	**β**	***SE***	***t***	***p***			**β**	***SE***	***t***	***p***	
Intercept	1.098	0.015	73.68	<0.001		Intercept	1.004	0.015	65.55	<0.001	
Test phase	−0.069	0.022	−3.09	0.002		Target upper left	−0.102	0.020	−4.98	<0.001	
Target upper left	−0.099	0.015	−6.78	<0.001		Diagonal alignment	0.113	0.020	5.77	<0.001	
Diagonal alignment	0.122	0.020	6.23	<0.001		Age	−0.004	0.002	−2.36	0.020	
Processing speed	0.002	0.001	2.13	0.034		Target upper left × diagonal	0.133	0.024	5.48	<0.001	
Test phase × target upper left	0.066	0.025	2.63	0.009							
Test phase × diagonal alignment	0.059	0.025	2.34	0.020							
**Random effects**		**Variance**	***SD***	**Corr**	**Corr**	**Random effects**		**Variance**	***SD***	**Corr**	**Corr**
Subject	Intercept	0.003	0.055			Subject	Intercept	0.003	0.057		
	Test phase	0.001	0.024	−0.738			Test phase	0.003	0.058	−0.187	
	Diagonal	0.010	0.101	−0.150	0.778		Diagonal	0.007	0.084	−0.300	−0.544
							Lower left	0.003	0.058		
							Upper left	0.005	0.069	−0.153	
Residual		0.096	0.310			Residual		0.120	0.346		

The age group comparison showed a significant effect of phase (beta = −0.137, *SE* = 0.045, *t* = −3.03, *p* = 0.002), indicating statistical learning in the group of younger adults, who were placed on the intercept. This effect of phase was modified by age group (beta = 0.125, *SE* = 0.061, *t* = 2.06, *p* = 0.039), suggesting that older adults learned less than younger adults and (given the almost equal beta values) that older adults were not affected by removal of the underlying regularities. This interaction between age group and phase tended to be less pronounced in diagonal trials (beta = −0.070, *SE* = 0.036, *t* = −1.91, *p* = 0.056). A fixed effect of age group indicated that, overall, older adults showed a lower facilitation score than younger adults (beta = −0.189, *SE* = 0.044, *t* = −4.28, *p* < 0.001). This effect of age group was influenced by both control variables. That is, the difference in facilitation score between younger and older adults was less distinct in diagonal (beta = 0.065, *SE* = 0.025, *t* = 2.54, *p* = 0.011) and in upper left trials (beta = 0.060, *SE* = 0.027, *t* = 2.25, *p* = 0.025). As expected, facilitation scores were higher in diagonal trials (beta = 0.120, *SE* = 0.019, *t* = 6.33, *p* < 0.001) and lower in trials, in which the first target appeared upper left (beta = −0.100, *SE* = 0.020, *t* = −4.98, *p* < 0.001). Moreover, the effect of phase was modified by both target position and by target alignment, implying that effects of statistical learning were less pronounced in diagonal trials (beta = 0.062, *SE* = 0.027, *t* = 2.29, *p* = 0.022) and in trials with an upper left target (beta = 0.064, *SE* = 0.027, *t* = 2.38, *p* = 0.017). The random slope structure indicated that participants differed in the degree to which they were affected by target position and by target alignment.

In the younger adults, the best-fitting model showed a significant effect of phase: the facilitation score of younger adults was lower in the test phase than at the end of the exposure phase, indicating that younger adults were affected by removing the underlying regularities. However, none of the individual listener characteristics interacted significantly with test phase, suggesting that amount of statistical learning was not associated with any of the selected measures of cognitive or linguistics abilities. Only processing speed showed a significant fixed effect on facilitation score, indicating that participants with higher processing speed had higher facilitation scores at the end of the exposure phase. As expected, facilitation scores were lower if the first target was displayed upper left and higher if targets were aligned diagonally. Both effects modulated learning in the anticipated direction: the effect of statistical learning was smaller in diagonal trials and in trials in which the first target was displayed upper left. In addition to the random slope of phase, the maximal random slope structure included random effects of first target position and target alignment on participant. Inclusion of these effects suggests that younger participants differed in the degree to which they were affected by target alignment, that is, whether they had to move the cursor horizontally or diagonally. Removing the random slope of phase within subject from the maximal random slope structure did not result in a significant loss in model fit, indicating that the amount of statistical learning did not differ considerably among younger adults (see Figure 3C).

Overall, older adults showed no significant effect of test phase, suggesting that they generally did not pick up the subtle regularities in the input. Age was the only individual background measure that predicted performance: the older the participants were, the lower was their facilitation score at the end of the exposure phase. In older adults, facilitation score was mainly influenced by the control variables. That is, diagonal alignment of targets enhanced facilitation scores and upper left position of the first target decreased facilitation benefit. A significant interaction between target position and target alignment indicated that effects of one control variable were modified by the other control variable: the effect of the first target being located upper left was smaller when participants could make a diagonal mouse movement to the second target, respectively, the benefit in facilitation score based on a diagonal movement was decreased in case the first target was displayed in the upper left corner of the screen. The maximal random slope structure showed that older adults differed in the degree to which they were affected by changes in target position (random slope of first target position within subject) and target alignment (random slope of first target position within subject). However, in modeling the statistical learning data of the older adults, we had kept in a random slope of phase to allow that participants may vary in how much their performance was affected by removing the regularities (we also needed this random slope parameter as the individual measure of statistical learning). Importantly, inclusion of this random effect of phase did not increase the model fit, implying that older participants did not differ much in their sensitivity to statistical regularities: they were all relatively insensitive to the probabilistic sequence information. Note that older adults continued to show increased facilitation throughout the exposure phase (cf. Figure 3A). As their performance was unaffected by the removal of the underlying regularities in the test phase, this suggests that the improvement over block in older adults reflects effects of task learning rather than effects of statistical learning.

### Perceptual learning

As we wanted to include statistical learning performance as a predictor in the analyses of the perceptual learning data alongside the auditory and cognitive measures, we checked for intercorrelations between statistical learning ability and other individual background measures. In the older adults, no correlations were observed. In the younger adults, intercorrelations between statistical learning performance and both working memory (*r* = −0.263, *p* = 0.044; rho = −0.297, *df* = 57, *p* = 0.022) and information processing speed (*r* = −0.279, *p* = 0.032; rho = −0.223, *df* = 57, *p* = 0.089) were significant: more learning was associated with better working memory and with higher processing speed.

**Figure 4:**
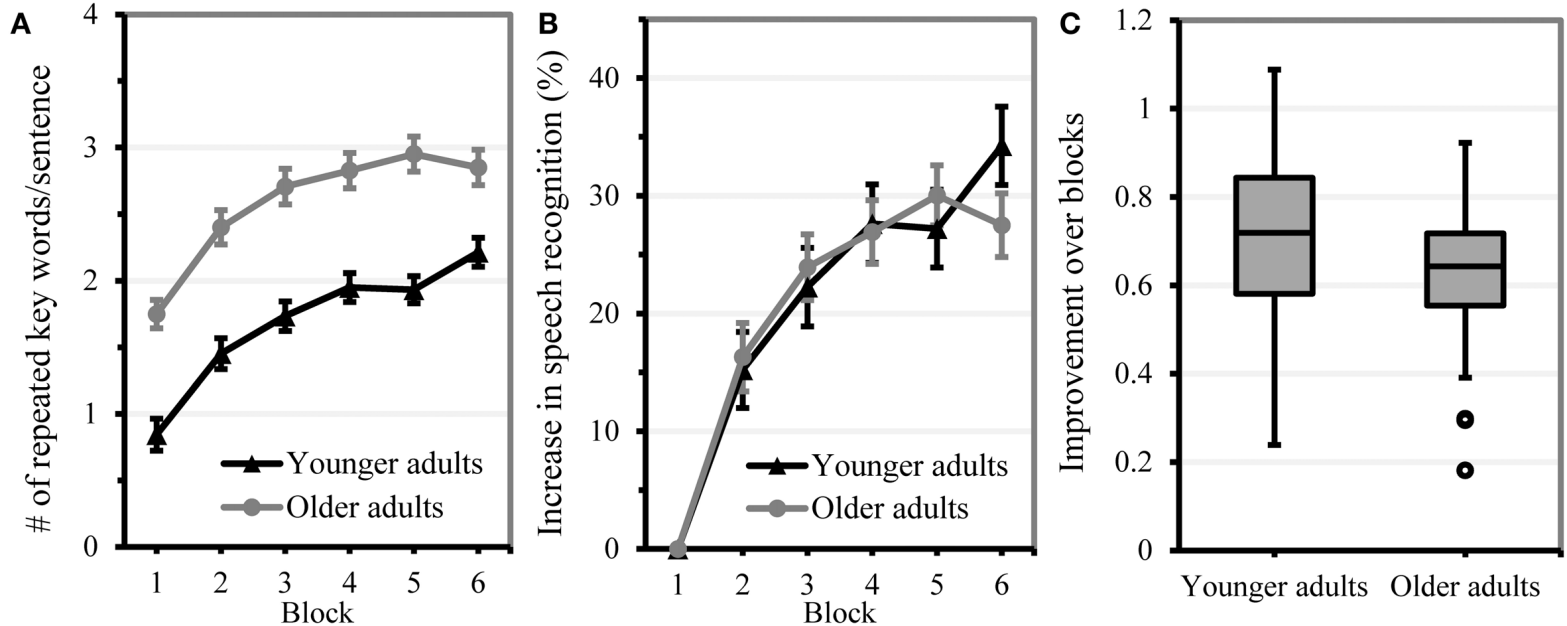
**Performance on the perceptual learning task**. Error bars indicate two standard errors from the mean. **(A)** Mean improvement in speech understanding per age group over block. **(B)** Improvement in speech understanding performance (in %) relative to baseline level. **(C)** Box plot of perceptual learning performance in younger and older adults (individual block slopes from the statistical model). More positive slopes reflect more learning.

In Figure 4, the average recognition score per block is displayed to illustrate perceptual learning of the noise-vocoded speech within age group. Moreover, Figure 4 shows the range of perceptual learning that could be observed within each age group. Although younger adults were presented with a more difficult noise-vocoding condition (4 bands) than older adults (5 bands) and showed a lower starting performance, both age groups showed similar progress in perceptual learning. This indicates that speech conditions were appropriately selected to elicit sizeable and comparable amounts of improvement over the course of exposure in both age groups. Estimates of the best model to predict sentence identification performance within each age group are displayed in Table 5.

**Table 5:** **Statistical models for sentence identification performance of younger and older adults in the perceptual learning task**.

**Fixed effects**	**Younger adults**				**Fixed effects**	**Older adults**			
	**β**	***SE***	***t***	***p***		**β**	***SE***	***t***	***p***
Intercept	0.917	0.155	5.90	<0.001	Intercept	1.897	0.122	15.60	<0.001
Block	0.703	0.043	16.41	<0.001	Block	0.624	0.037	16.73	<0.001
Statistical learning	6.649	8.966	0.74	*n.s*	Hearing	−0.029	0.005	−6.23	<0.001
Vocabulary	−0.921	1.255	−0.73	*n.s*.	Processing speed	0.011	0.004	2.46	0.017
Block × statistical learning	−9.223	4.058	−2.27	0.023	Age	0.004	0.011	0.38	*n.s*.
Block × vocabulary	1.489	0.568	2.62	0.009	Block × Age	−0.017	0.007	−2.53	0.012
**Random effects**		**Variance**	***SD***	**Corr**	**Random effects**		**Variance**	***SD***	**Corr**
Subject	Intercept	0.436	0.660		Subject	Intercept	0.122	0.350	
	Block	0.049	0.221	−0.821		Block	0.040	0.201	−0.509
Item	Intercept	0.912	0.955		Item	Intercept	2.770	1.664	
						Hearing	0.000	0.015	
						Age	0.001	0.002	−0.887
Residual		1.284	1.133		Residual		1.307	1.143	

*n.s. = p > 0.05*.

The age group comparison showed a significant effect of block (beta = 0.710, *SE* = 0.034, *t* = 20.78, *p* < 0.001) that was not modified by age group (beta = −0.071, *SE* = 0.046, *t* = −1.56, *p* = 0.120), indicating that both age groups showed a similar amount of perceptual learning over the course of the experiment^3^. As older adults were presented with an easier condition (5 instead of 4 band vocoded speech), a fixed effect of age group showed that older adults repeated more key words correctly than younger adults (beta = 0.971, *SE* = 0.103, *t* = 9.41, *p* < 0.001). Our results suggest that we were successful in providing older and younger adults with a starting level that allowed for a comparable amount of perceptual learning within both age groups.

In younger adults, none of the predictor measures showed a fixed effect, suggesting that none of the predictor measures could be used to predict initial speech recognition performance (i.e., for block 1 performance, being on the intercept). The best fitting model showed that younger participants identified more keywords correctly with increasing exposure over blocks, indicating that they generally adapted to noise-vocoded speech. This effect of perceptual learning was modified by statistical learning ability: the more participants had picked up the implicit regularities in the statistical learning task (and thus the steeper their drop in performance in the test phase), the more they improved in understanding noise-vocoded speech. This result provides first evidence that perceptual learning and statistical learning are associated. Further, the effect of perceptual learning was modified by vocabulary knowledge: younger adults who had greater vocabulary knowledge showed faster speech adaptation over blocks, underscoring the involvement of linguistic knowledge in perceptual learning of speech. Note that we had excluded an interaction between block and processing speed during the modeling process, as its inclusion led only to a marginal improvement of model fit. This marginal interaction suggested that higher processing speed tended to be associated with faster adaptation. The maximal random slope structure included the effect of block within subject. Removing this effect from the maximal random slope structure reduced the model fit significantly, indicating that individuals differed considerably in perceptual learning ability. As random slopes of individual predictor measures within items did not improve the model fit, this indicated that the effects of predictor measures could be generalized across sentences.

In the older adults, initial sentence identification performance was associated with hearing sensitivity and processing speed: hearing loss considerably affected initial speech understanding, whereas those with higher processing speed showed better initial speech recognition performance. Like the younger adults, older adults showed perceptual learning of noise-vocoded speech, which was indicated by a significant improvement in identification performance over blocks. This block effect was modified by age, indicating that older adults within the older age group improved less over the course of exposure than younger older adults. As age in the older adult sample was intercorrelated with processing speed and hearing sensitivity (see intercorrelations in Table 2), we also investigated whether either variable would have surfaced as a predictor for adaptation if we left out age. The variance in amount of perceptual learning that was assigned to age was not taken over by any of the other predictors included in the analysis. This indicates that the effect of age explains unique variance in perceptual learning performance that is not captured by the included cognitive and perceptual predictors. Importantly, statistical learning ability did not facilitate the amount of improvement over the course of the experiment. The maximal random slope structure included effects of age and hearing sensitivity on item, suggesting that the effects of age and hearing sensitivity on recall of noise-vocoded sentences differed across sentences. That is, hearing and age affected speech understanding of some sentences more than of others, in addition to the general impact these predictors had on sentence recall. Moreover, inclusion of the random effect of block within participant significantly improved the model fit, implying that older participants differed in their improvement to understand noise-vocoded speech over the course of exposure.

## Discussion

This study investigated the contribution of general cognitive abilities to listeners' capacity to adapt to novel speech conditions. In order to gain more insight into individual abilities associated with adaptation to unfamiliar speech across the life span, we tested both younger and older adults. Specifically, we aimed to test the hypothesis that listeners' improvement in understanding unfamiliar types of speech could be predicted from individual differences in statistical learning ability and in general cognitive skills.

The ability to implicitly learn has been argued to remain stable over the life span (Midford and Kirsner, 2005). In line with this, several studies reported that older adults are sensitive to probabilistic sequences (Salthouse et al., 1999; Negash et al., 2003; Simon et al., 2011; Campbell et al., 2012) and found the ability to adapt to novel speech conditions to be preserved in older adults (Peelle and Wingfield, 2005; Golomb et al., 2007; Adank and Janse, 2010; Gordon-Salant et al., 2010). Our findings support the notion that perceptual learning ability remains stable over the life span, as both younger and older listeners showed significant improvement in understanding noise-vocoded speech over exposure. Moreover, the observed amount of learning was comparable in both age groups. This suggests that older adults can reach the same amount of perceptual learning as younger adults given better starting level intelligibility. However, only younger adults were sensitive to statistical regularities in the input. As we found a significant learning by age group interaction, this indicated age-related declines in the ability to detect statistical regularities if visual sequences are presented quickly.

Possibly, certain aspects of our statistical learning task may be responsible for the absence of a statistical learning effect in older adults. In particular, we had incorporated an inter-target interval of 500 ms (following Salthouse et al., 1999) between both clicks within a trial to allow for prediction effects, even in older adults with slower processing. As we tested statistical learning in a speeded computer mouse task, and movement control on computer mouse tasks is reduced in older adults (Smith et al., 1999), the implemented inter-target interval may have been too short for older adults to show prediction effects. Moreover, to prevent associations between both measures of implicit learning due to modality-specific processing, we chose for a rigorous test of the association between the two types of learning by testing statistical learning ability in a non-auditory (i.e., visual) domain with non-linguistic stimuli. As older adults were able to implicitly learn in the auditory task, it may be argued that we did not observe implicit learning in the visual paradigm due to age-specific modality effects. In both implicit learning tasks, task-relevant information was presented sequentially (i.e., speech unfolding over time in the auditory task and successive highlighting of targets in the visual task). Visual stimuli have been shown to have less salient temporal relations than auditory stimuli (Kubovy, 1988). Consequently, auditory learning is superior to visual learning in sequence learning tasks (Conway and Christiansen, 2005). Additionally, a recent study found that statistical learning performance is decreased if visual stimuli are presented at a fast rate (Emberson et al., 2011). Although stimuli presentation in our statistical learning task was not timed as it depended on participants' performance speed (i.e., participants who clicked faster, saw visual stimuli shorter), the time pressure induced by the speeded task, as well as relatively fast and sequential presentation of visual stimuli, may have interfered with statistical learning performance in older adults. That is, results of the current study suggest that older adults' statistical learning ability is affected if fast, sequential processing of visual stimuli is required. However, as previous studies have shown that older adults remain sensitive to probabilistic information in the input (Salthouse et al., 1999; Negash et al., 2003; Simon et al., 2011; Campbell et al., 2012), our failure to observe statistical learning in older adults should not be taken as evidence that older adults are generally insensitive to probabilistic information in the input, or that probabilistic information in the input is generally unimportant for perceptual learning in older adults. Obviously, further research is required to investigate possible links between statistical and perceptual learning in a setting where older adults do show both types of learning.

Overall, limited variability could be observed on the measure of statistical learning ability in both age groups and the amount of individual statistical learning could not be explained by individual differences in cognitive or linguistic abilities in our analyses. However, note the correlations between statistical learning on the one hand and speed and working memory on the other hand in the younger adults. These correlations suggest that, despite relatively little variation in statistical learning, there was some systematicity in younger adults' statistical learning differences. In contrast, participants showed great variability in the amount of adaptation to degraded speech and individual differences in learning to understand noise-vocoded speech could be associated with listeners' cognitive abilities. This finding supports the claim of the RHT that perceptual learning is a top–down guided process, implying that higher cognitive processes are indeed involved in the top–down search to identify task-relevant cues in the input. However, links between cognitive abilities and perceptual learning performance seem to undergo age-related changes, as different associations between perceptual learning ability and cognitive measures emerged in younger and older adults.

In younger adults, initial performance in identifying noise-vocoded speech was not predicted by general cognitive or linguistic abilities. However, differences in the amount of improvement over the course of exposure were associated with individual sensitivity to probabilistic information and with individual vocabulary knowledge. In line with our hypothesis, our results suggest that adaptation to novel speech conditions and statistical learning share mechanisms of implicit regularity detection. Our results contribute to earlier literature indicating a relationship between statistical learning performance and individual differences in language processing (Misyak et al., 2010a). As statistical learning was tested using visual and non-linguistic stimuli, this suggests that general abilities, that are neither modality-specific nor specific for language processing, drive this association.

As argued in the Introduction, the link between statistical learning and perceptual learning in speech can be twofold. On the one hand, statistical and perceptual learning may be associated as they draw on the same underlying abilities. Our findings do not support this “mediation account”: the observed association between perceptual learning in speech and statistical learning performance does not seem to be mediated by the specific cognitive abilities tested in the current study. On the other hand, perceptual learning processes may directly rely on statistical properties in the input. In novel speech conditions, perceptual learning may be facilitated by sensitivity to statistical properties as language itself conveys probabilistic information e.g., in terms of phonotactic (Vitevitch et al., 2004) and transitional probability (Thompson and Newport, 2007). Listeners have been shown to make use of this probabilistic information to segment speech streams into words (Saffran et al., 1997). In the framework of the RHT, listeners who are more sensitive to statistical regularities may, hence, be faster in identifying subunits (e.g., words) in novel speech input, thereby facilitating faster access to high-level representations. Moreover, the information that is transferred from lower to higher levels of the hierarchy may itself be probabilistic in nature. Recent theories in visual perceptual learning argue that the process of input reweighting is based on such probabilistic decisions (e.g., Petrov et al., 2005; Zhang et al., 2010). For example, assuming that the information that is conveyed from lower levels to higher levels is normally distributed and that the mean of the distribution resembles the most relevant input, each incoming input could be reweighted based on its relative distance from the mean, with distance serving as index of informational relevance (Zhang et al., 2010). First evidence that probabilistic information may be encoded in the input from lower to higher hierarchical levels comes from a study in which neuronal network models that relied on probabilistic inferences could explain neurophysiological changes in early sensory areas in visual perceptual learning tasks that could not be accounted for by other models (Bejjanki et al., 2011).

The finding that improvement in understanding noise-vocoded speech in younger adults is predicted by participants' vocabulary size confirms the link between increased lexical knowledge and success in perceptual learning that has previously been reported in adapting to novel-accented speech (Janse and Adank, 2012). Thus, lexical knowledge is not only associated with adaptation to linguistic degradations, e.g., systematic phonological deviations in how a foreign-accented speaker pronounces words, but also relates to perceptual learning of acoustically degraded speech. Previous research has shown that younger and older individuals with higher scores on vocabulary tests also show better performance on measures of verbal fluency (e.g., Kemper and Sumner, 2001; Hedden et al., 2005). Thus, individuals with greater vocabulary knowledge may be more efficient processors of linguistic information (Kemper and Sumner, 2001), and linguistic knowledge may improve perceptual learning in speech by facilitating access to higher-level representations. As access to higher-level representations aids sublexical retuning by enabling and guiding top–down search processes (Ahissar and Hochstein, 2004), effects of lexical knowledge should in fact arise irrespective of type of systematic speech degradation. Given that Janse and Adank (2012) observed a relationship between vocabulary knowledge and adaptation (to accented speech) in older adults, this raises the question why older adults' perceptual learning performance was not predicted by their linguistic knowledge here. Older adults outperformed younger adults on the measure of lexical knowledge, but note that older adults also showed relatively little variation on the vocabulary test (see Section Performance on Background Measures). Consequently, there was less room to relate lexical knowledge to individual differences in perceptual learning ability in older adults than in younger adults. We checked the variation for older adults' vocabulary scores in the sample of Janse and Adank (2012) (coefficient of variation = 10.3%), which was close to the variation we now observed in the younger adults. Therefore, variation in older adults' vocabulary scores in the current study may have indeed been insufficient to predict perceptual learning.

In the older adult group, listeners' starting level in understanding noise-vocoded speech was associated with higher processing speed and affected by hearing loss, whereas listeners' age predicted how well they adapted to the novel speech condition. That is, younger listeners in the group of older adults showed more learning than older–older listeners. This effect of age had unique explanatory power that was not captured by the included cognitive and perceptual predictors. This finding seems to be consistent with previous research which reported declines in the general identification of noise-vocoded speech with increasing age that were independent of hearing sensitivity (Souza and Boike, 2006; Sheldon et al., 2008) and which may have reflected limited improvement over exposure. Importantly, the current design allowed us to differentiate between effects of individual predictors on both starting level speech identification performance and on amount of perceptual learning. Thus, our results complement earlier findings, suggesting that hearing loss affects initial recognition of noise-vocoded speech, whereas age-related deficits specifically constrain improvement in adaptation to a novel speech input. Younger adults generally outperform older adults when being exposed to the same speech degradation (Peelle and Wingfield, 2005; Sheldon et al., 2008). Importantly, providing younger and older adults with the same speech degradation also has consequences for age groups' ability to improve their performance over exposure (cf. our pilot result data discussed in Section 3.3). In order to have similarly large amounts of perceptual learning for the two age groups, older adults have to be presented with an easier condition than younger adults (Golomb et al., 2007), which was also done in the current study. It is unclear, however, what the age effect on perceptual learning ability among the older adults reflects. A possible account may come from recent studies reporting that coherence between activated brain regions is decreased in older adults (Andrews-Hanna et al., 2007; Peelle et al., 2010), relative to younger adults. Importantly, these deteriorations in connectivity were associated with declines in speech understanding performance under difficult listening conditions (Peelle et al., 2010) and with poorer performance on cognitive tasks (Andrews-Hanna et al., 2007). In the framework of the RHT, we may speculate that a reduced coordination between neuronal regions may hinder effective information flow between hierarchical levels, thereby constraining processes of input reweighting. Consequently, this decreased information flow would then impede modifications to the lower-level representations. Thus, an age-related decrease in the ability to coordinate activity between brain regions may affect adaptation to challenging novel speech input.

In short, our results suggest that individual differences in general cognitive and linguistic abilities can explain listeners' variability in adaptation to noise-vocoded speech, thereby highlighting the involvement of listener-based abilities in perceptual learning. As noise-vocoded speech simulates the auditory signal of a cochlear implant, findings of the current study may provide valuable insights for aural rehabilitation in younger and older adults. Amount of adaptation over the course of exposure was specifically associated with vocabulary knowledge and with individuals' sensitivity to probabilistic regularities. These combined results emphasize the importance of pattern recognition and linguistic knowledge for perceptual learning and adaptation in speech processing.

## Author contributions

Thordis M. Neger was intensively involved in literature search, research design, experiment preparation, data collection, data analyses, data interpretation, and article preparation. Esther Janse was intensively involved in research design, data analyses, data interpretation, and article preparation. Toni Rietveld was involved in data analyses and article preparation, and contributed to the research design. All authors approved the final article.

### Conflict of interest statement

The Review Editor Frank Eisner declared to the Guest Associate Editor as being affiliated to the same institution as the authors prior to accepting the review assignment, and the disclosure made was deemed sufficient in this case. The authors declare that the research was conducted in the absence of any commercial or financial relationships that could be construed as a potential conflict of interest.

## Footnotes

^1^Note that we ran a second analysis in which we kept the original index of block. The analysis resulted in the same best models and showed the same effects as the models reported here. However, models that included the log-transformed index of block showed an increased model fit, indicating non-linear learning behavior.

^2^Note that the drop in performance that can be observed in the younger adults during the exposure phase (see Figure 3A, blocks 5 and 6) is not significant (beta = −0.018, *SE* = 0.010, *t* = −1.71, *p* = 0.088). This suggests that there was no general drop in performance across the group of younger adults. Likewise, the spread of the individual slope data (*M* = −0.018, *SD* = 0.019, Min = −0.078, Max = 0.027) also includes positive slope values (indicating improvement, rather than decreased performance). Moreover, a paired samples t-test shows that the size of the unexpected drop in the exposure phase is significantly smaller than the drop in the test phase that is considered to reflect learning (*t* = 17.91, *df* = 58, *p* < 0.001).

^3^Note that we also performed an age group comparison including the data of the pilot study. In this analysis, we compared younger adults' performance on the 4 bands speech (placed on the intercept) to older adults' performance across the different band conditions (i.e., 4 bands, 5 bands and 6 bands). In the 4 band condition, younger adults showed a higher starting performance than older adults (beta = −0.639, *SE* = 0.160, *t* = 3.99 and *p* < 0.001). More importantly, in the 4 band condition, we found an interaction between age group and improvement over blocks. That is, older adults showed a significantly smaller effect of block and, hence, less learning, than younger adults (beta = −0.098, *SE* = 0.026, *t* = −3.80 and *p* < 0.001). This result emphasizes that it is not possible to elicit similar amounts of perceptual learning in the two age groups by presenting younger and older adults with the same signal degradation condition.
