# Supplementary material for: Relationship between perceptual learning in speech and statistical learning in younger and older adults
Source: Front Hum Neurosci. 2014 Sep 1;8:628. doi: 10.3389/fnhum.2014.00628 (PMC4150448; doi:10.3389/fnhum.2014.00628)
Supplement: Supplementary file 1 [file DataSheet1.DOCX]

Appendix

A1. Sentences from Versfeld et al. (2000) that were used in the perceptual learning task

|  | **Dutch** | **English translation** |
| --- | --- | --- |
|  | Practice sentences |  |
| 1 | Zijn vrouw is naar de winkel gegaan | His wife went to the shop |
| 2 | Midden op het grasveld wordt gedanst | They dance in the middle of the lawn |
| 3 | Ze trekt er een vies gezicht bij | She distorted her face in disgust |
| 4 | Op de foto lijken ze echt | They look authentic on the picture |
| 5 | Men wil meer aan onderzoek gaan doen | One wants to do more research |
|  | Test sentences |  |
| 1 | Het gesprek loopt via tolken | The conversation goes via an interpreter |
| 2 | Aan zijn voeten zit een hondje | A dog sits at his feet |
| 3 | Mijn kinderen zitten hier op school | My children go to school here |
| 4 | In de bomen fluiten de vogels | Birds sing in the trees |
| 5 | In het weekend maakt hij het ontbijt | He makes breakfast on weekends. |
| 6 | In de verte ligt een kasteel | There is a castle in the distance |
| 7 | Hij haalde de kinderen van school | He picked the children up from school. |
| 8 | Ik eet beschuit met aardbeien | I eat rusk with strawberries |
| 9 | Hij trekt zijn muts over zijn voorhoofd | He pulls his hat over his forehead |
| 10 | De sneeuw glinstert in het maanlicht | The snow glistens in the moonlight |
| 11 | In de verte grazen schapen | Sheep graze in the distance |
| 12 | Mijn auto staat op de kade | My car is by the wharf |
| 13 | De regen klettert op de voorruit | The rain patters against the windshield |
| 14 | Zijn vader zit in de kamer | His father sits in the room |
| 15 | Het water spettert op haar benen | The water splashes on her legs |
| 16 | De toerist kiest voor zekerheid | The tourist opted for safety |
| 17 | De tranen komen in zijn ogen | Tears came to his eyes |
| 18 | De adem stokte in mijn keel | My breath stuck in my throat |
| 19 | Zijn t-shirt plakte tegen zijn borst | His t-shirt sticked to his chest |
| 20 | De pannen staan op het fornuis | The pots are on the stove |
| 21 | De regen striemde in mijn gezicht | The rain lashed me in the face |
| 22 | De kinderen spelen in de tuin. | The children play in the garden |
| 23 | Op de tafel stond een koffiepot | A coffee pot is on the table |
| 24 | Ze verkopen sokken op de markt | They sell socks on the market |
| 25 | Hij kent de regels van het spel | He knows the rules of the game |
| 26 | In het duister nadert een trein. | A train approaches in the dark |
| 27 | Ik koop een kaartje aan het loket | I buy a ticket at the ticket office |
| 28 | Het gereedschap ligt in de schuur | The tools are in the shed |
| 29 | Er hing een briefje op de deur | There was a note on the door |
| 30 | Zijn vrouw speelde op de piano | His wife played the piano |
| 31 | De chauffeur kijkt op zijn horloge | The driver looked at his watch |
| 32 | Het gebeurt in een razend tempo | It happens at breakneck speed |
| 33 | Ik kijk naar een leuk programma | I watch a nice program |
| 34 | Ze dronk uit een zilveren beker | She drank from a silver mug |
| 35 | Ik liep vlug naar de bushalte | I went quickly to the bus stop |
| 36 | Ze houden van lekker eten | They are fond of good food. |
| 37 | Hij blijft op zijn eigen kantoor | He stays in his own office |
| 38 | Uitgeput zit hij aan tafel | He sits at the table, exhausted. |
| 39 | Hij kijkt naar zijn blote voeten | He looks at his bare feet |
| 40 | Ze schrikt van haar eigen woorden | She is shocked by her own words |
| 41 | Ze woont in een grote woning | She lives in a big house |
| 42 | Zijn pet is te groot voor zijn hoofd | His hat is too large for his head |
| 43 | Hij woont in een piepklein huisje | He lives in a tiny house |
| 44 | Hij vertrok op een oude fiets | He left on an old bike |
| 45 | Ze woont tijdelijk bij haar tante | She lives at her aunt's place temporarily |
| 46 | Samen wachten we op de bus | We wait for the bus together. |
| 47 | Ik wandelde vaak met de hond | I used to walk the dog frequently |
| 48 | Hij komt uit een andere cultuur | He has a different cultural background |
| 49 | Hij helpt vaak in de vakanties | He often helps during the holidays |
| 50 | Ik keek hem recht in zijn gezicht | I looked him straight in the face |
| 51 | Je raakt helemaal uit je ritme. | You lose your rhythm completely |
| 52 | Hij stond graag in de schijnwerpers | He liked to be in the spotlights |
| 53 | Genietend drinkt hij van zijn thee | He enjoyed to drink his tea |
| 54 | Hij koos voor een andere aanpak | He opted for a different approach |
| 55 | Hij verbergt zijn hand achter zijn rug | He hides his hands behind his back |
| 56 | Ik verheug me erg op het feest | I am looking forward to the party |
| 57 | Het plan spreekt tot de verbeelding | The plan appeals to one's imagination |
| 58 | Op de vensterbank staat een plant | There is a flower on the windowsill |
| 59 | In de keuken staat een ijskast | There is a fridge in the kitchen |
| 60 | De actie duurt tot oktober | The campaign lasts until October |
